# Can a rational design for metronomic chemotherapy dosing be devised?

**DOI:** 10.1038/sj.bjc.6602474

**Published:** 2005-04-20

**Authors:** A Maraveyas, T Lam, J W Hetherington, J Greenman

**Affiliations:** 1 Department of Academic Oncology, The Princess Royal Hospital, Saltshouse Road, Hull HU8 9HE, UK; 2 Department of Surgery, University of Hull, Cottingham Road, Hull HU6 7RX, UK; 3 Department of Urology, Castle Hill Hospital, Castle Road, Hull HU16 5JQ, UK

**Sir**,

An emerging strategy for refractory adult solid malignancies is to manage cancer as a chronic but stable disease state, where the total tumour burden is kept at the lowest possible level. One of the most likely strategies to achieve this would seem to be the targeting of neoangiogenesis. Recently, a new paradigm has emerged based on the targeting of tumour vasculature rather than tumour parenchyma with low-dose but long-term, continuous, chemotherapy using classical cytotoxic agents. The term ‘metronomic dosing’ was first used by Hanahan *et al* (2000). For further details on metronomic chemotherapy (MC), see the recent, extensive, review by Kerbel and Kamen (2004).

While intuitively there should be no disagreement with what constitutes a metronomic schedule, our major concern relates to the dosing levels. The starting point for conventional dosing is empirical, for example, 
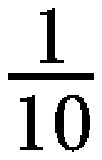
th of the LD_50_ of a preclinical, animal-based, toxicity study. From then on, the dose for human use is based on the Phase I study. The starting dose for MC is equally empirical with the major proponents commencing clinical or preclinical trials at 10–33% of conventional doses; no further effort to define this dose is made! The historical variability of clinical response to chemotherapy and the concern about intertrial reproducibility of results will be compounded by the lack of dose uniformity. It is hard enough correlating responses among different conventional dosing schemes, for example, taxanes in ovarian cancer; it will be even more difficult if the dosing scheme has little rhyme or reason justifying its selection. Given the large number of drugs with potential antiangiogenic properties when given metronomically, the further development of combination treatments will become even more contentious. We think that a strategy to deal with this problem is necessary given that extreme empiricism could breed scepticism which in turn would significantly delay progress in this field. Fortunately, we would suggest that there are already enough data to allow for a principled approach to metronomic dosing.

Reflecting conventional design, the metronomic Phase I trial methodology that we propose is based on the following principles:

- Choice of initial dose and dosing schedule.
- Dose ranging.
- End points.

- While conventional dosing schedules often have bewildering complexity, only two schedules would be relevant for metronomic delivery (MetS), and these are daily oral and continuous infusion schedules. Pharmacokinetic studies of drug and metabolites given in these continuous schedules should be undertaken as per the conventional approach. A logical extension of this is that all new drugs (especially oral agents) developed should also have a continuous dosing schedule in their Phase I development portfolio.
- We would argue that dose ranging is essential given that there is very good evidence *in vitro* that different agents have varying capacities to inhibit the growth of endothelial cells at different doses. Work from our laboratory (Figures 1 and 2) clearly shows that for similar exposure times and conditions, the effects of an alkylating agent (temozolomide) are different to those of an antimitotic agent (estramustine) on human umbilical vein endothelial cell (HUVEC) growth. Furthermore, even at these low drug doses, we demonstrate a clear dose–response curve. We would therefore propose that the highest dose that can be given in a metronomic manner would be likely to have the greatest effect on the endothelium.
- The crux of the matter is defining the ‘dose limit’ related to the second principle articulated above, that is, the clinical metronomic dose (MetD). Bone marrow suppression is the single most potent, clinically preventable, proangiogenic stimulus related to conventional treatments. Release of haematopoietic stem cells including endothelial progenitor cells (CEP) from the bone marrow into the circulation in response to chemotherapy, cytokine stimulation or irradiation was first documented more than three decades ago (reviewed in To *et al*, 1997). Animal models have verified that CEP play a major role in tumour neoangiogenesis, and that there is a major difference in CEP mobilisation for the same agent (cyclophosphamide) when comparing conventional and metronomic dosing regimes (Bertollini *et al*, 2003). We therefore propose that MetD should be the highest dose that can be delivered in a metronomic schedule without causing clinical bone marrow perturbation, assuming that there are no other dose-limiting toxicities that appear before bone marrow disruption. Monitoring the full blood count, although practical, may be insensitive; therefore, research into developing assays of bone marrow stem cell activation circulation is likely to produce more useful tools in the near future.

In conclusion, scheduling of MC is arguably unequivocal; dosing, however, is poorly defined. We argue that even in the subclinical dose range, there is a dose–response effect on the endothelium, and that using an arbitrary 10–33% of the conventional dose is not always appropriate. Given that bone marrow suppression is a proangiogenic stimulus, we propose that the MetD should be the maximum dose that can be delivered in a metronomic schedule without detectable bone marrow perturbation. Such a rational strategy would allow MC dosing to be derived via a principled approach.

## Figures and Tables

**Figure 1:**
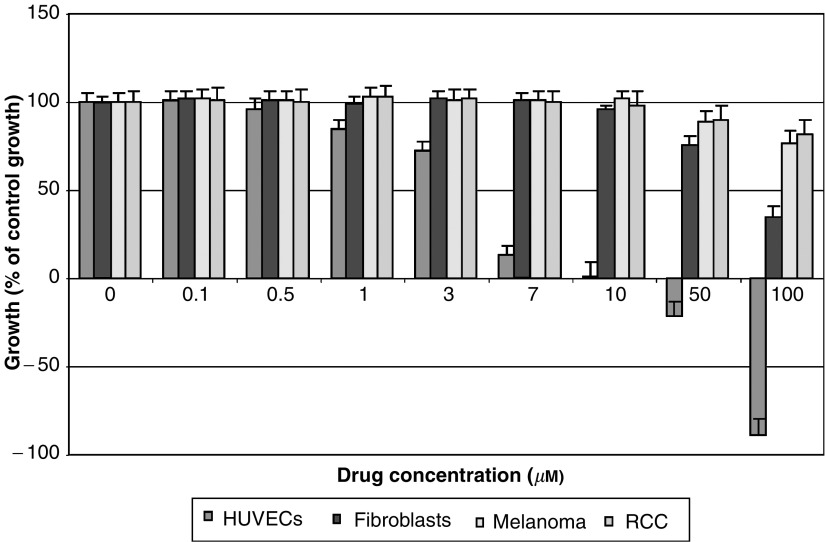
Endothelial cell-specific growth inhibition at low doses following 4-day continuous exposure to estramustine. Experiment conducted as quadruplicates and repeated at least twice. All cells, that is, HUVECs, human fibroblasts, human melanoma and human RCC cells were under passage 6 and grown as monolayers in 96-well plates and treated with either agent for 4 days, with daily replacement and replenishment of media. Cell proliferation assay performed via MTS assay with background-subtracted absorbance at 490 nm determined hourly for up to 4 h. Baseline cell growth=((cell no. of test at 4 days−cell no. of test prior to treatment)/cell no. of test prior to treatment) × 100%. The baseline growth of control, untreated, cells (i.e. control) is taken as 100% and growth of treated populations of cells is expressed as % of this control growth (mean±s.e.).

**Figure 2:**
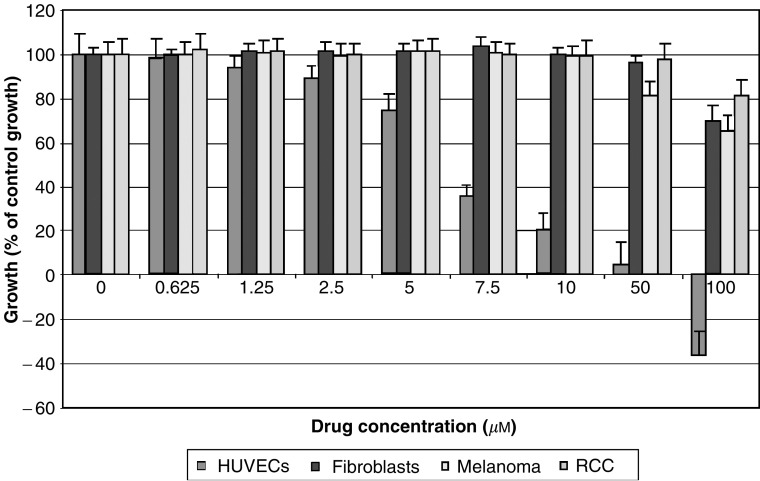
Endothelial cell-specific growth inhibition at low doses following 4-day continuous exposure to temozolomide. Experiment conducted as quadruplicates and repeated at least twice. All cells, that is, HUVECs, human fibroblasts, human melanoma and human RCC cells were under passage 6 and grown as monolayers in 96-well plates and treated with either agent for 4 days, with daily replacement and replenishment of media. Cell proliferation assay performed via MTS assay with background-subtracted absorbance at 490 nm determined hourly for up to 4 h. Baseline cell growth=((cell no. of test at 4 days−cell no. of test prior to treatment)/cell no. of test prior to treatment) × 100%. The baseline growth of control, untreated, cells (i.e. control) is taken as 100% and growth of treated populations of cells is expressed as % of this control growth (mean±s.e.).

## References

[bib1] Bertollini F, Paul S, Mancuso P, Monestiroli S, Gobbi A, Shaked Y, Kerbel RS (2003) Maximum tolerable dose and low-dose metronomic chemotherapy have opposite effects on the mobilization and viability of circulating endothelial progenitor cells. Cancer Res 63: 4342–4346 12907602

[bib2] Hanahan D, Bergers G, Bergsland E (2000) Less is more, regularly: metronomic dosing of cytotoxic drugs can target tumor angiogenesis in mice. J Clin Invest 105: 1045–1047 10772648 10.1172/JCI9872 PMC300842

[bib3] Kerbel RS, Kamen BA (2004) The anti-angiogenic basis of metronomic chemotherapy. Nat Rev Cancer 4: 423–436 15170445 10.1038/nrc1369

[bib4] To LB, Haylock DN, Simmons PJ, Juttner CA (1997) The biology and clinical uses of blood stem cells. Blood 89: 2233–2258 9116266

